# Application of Genetic Optimization Algorithm in Financial Portfolio Problem

**DOI:** 10.1155/2022/5246309

**Published:** 2022-07-15

**Authors:** He Li, Naiyu Shi

**Affiliations:** School of Sciences, Changzhou Institute of Technology, Changzhou 213000, China

## Abstract

In order to address the application of genetic optimization algorithms to financial investment portfolio issues, the optimal allocation rate must be high and the risk is low. This paper uses quadratic programming algorithms and genetic algorithms as well as quadratic programming algorithms, Matlab planning solutions for genetic algorithms, and genetic algorithm toolboxes to solve Markowitz's mean variance model. The mathematical model for introducing sparse portfolio strategies uses the decomposition method of penalty functions as an algorithm for solving nonconvex sparse optimization strategies to solve financial portfolio problems. The merging speed of the quadratic programming algorithm is fast, and the merging speed depends on the selection of the initial value. The genetic algorithm performs very well in global searches, but local search capabilities are insufficient and the pace of integration into the next stage is slow. To solve this, using a genetic algorithm toolbox is quick and easy. The results of the experiments show that the final solution of the decomposition method of the fine function is consistent with the solution of the integrity of the genetic algorithm. 67% of the total funds will be spent on local car reserves and 33% on wine reserves. When data scales are small, quadratic programming algorithms and genetic algorithms can provide effective portfolio feedback, and the method of breaking down penalty functions to ensure the reliability and effectiveness of algorithm combinations is widely used in sparse financial portfolio issues.

## 1. Introduction

In recent years, China's venture capital industry has developed rapidly, and the total amounts of venture capital enterprises (funds) and venture capital management funds are increasing. The increasing number and variety of securities investment not only greatly enrich the products of the financial market, but also become a backbone force in the financial market and play an important role in the stable development of the market. Securities investment is an important part of the operation of the securities market, and securities portfolio theory is one of the most important securities investment theories. Therefore, it is of great significance to establish an appropriate model and choose an effective algorithm to solve the portfolio problem. In order to avoid complex mathematical programming, many scholars use intelligent algorithms to solve portfolio problems [1].

Market investment has both the possibility of profit and the risk of loss. Portfolio is a commonly used way to avoid risk. The reasonable goal of building a portfolio should be to achieve the highest return portfolio as far as possible under a certain risk level, that is, an effective portfolio. In order to build a portfolio that can achieve the most effective goal, Markowitz model provides a clear training process—optimization. Optimization process is widely used in the process of asset allocation. Since the main asset types available are limited in the actual process, the process is operable [2].

Optimization models and algorithms are playing an increasingly important role in financial decision making. From asset allocation to risk management and option pricing to modeling, many financial mathematical problems can be effectively addressed through modern optimization methods. Optimization methods are a branch of applied mathematics. Mathematically, this means finding the minimum or maximum value of the objective function under certain conditions [3]. A typical optimization problem is to achieve the goal optimization by allocating different proportions of resources, such as cost minimization, profit maximization, and so on. Optimization method is a mathematical method to solve optimization problems. Traditional optimization methods mainly include branch definition method, background segmentation method, dynamic programming method, and other accurate algorithms. The absolute optimal solution can be obtained by accurate algorithm, but because of its large calculation scale, it is only suitable for solving small-scale problems and is not suitable for application in complex engineering problems with high-dimensional, multiobjective, and multiconstraints. On the contrary, heuristic algorithm refers to the method of searching according to empirical rules when solving problems, rather than solving according to determined steps. After the 1950s, scholars applied heuristic algorithms to practical complex engineering problems and obtained good feedback, which opened the research wave of heuristic algorithms [4].

The purpose of this article is to understand the effects of various methods of optimization in theory and practice. Therefore, we have chosen the M-V model, which is the simplest square error model.

M-V model, as shown in the following formulas:

(1) $$\begin{array}{c} \underset{x}{\min}\sigma^{2}=\frac{1}{2}x^{T}Vx, \end{array}$$

(2) $$\begin{array}{c} \text{s}\left\{\begin{array}{c} r^{T}x\geq \mu ,\\ e^{T}x=1,\\ L\leq x\leq U, \end{array}\right. \end{array}$$

where *V* represents the covariance matrix of each financial asset (in this case, stock), (*r*_1_, *r*_2_,…,*r*_*n*_)^*T*^ represents the rate of return of each stock, and *μ*=(*μ*_1_, *μ*_2_,…,*μ*_*n*_)^*T*^ represents the investment return expected by investors.

In the portfolio theory, the average value of the return on assets is used as the measurement index of the expected return on assets, and the variance of the return on assets is used as the risk measurement index of the portfolio [5].

## 2. Related Works

**Figure 1:**
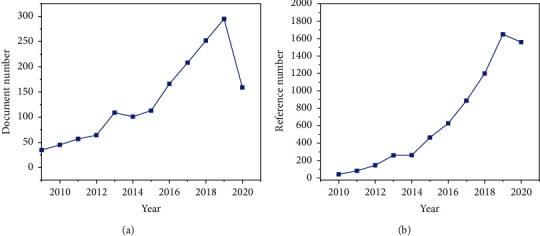
Publication and citation of portfolio optimization literature. (a) Number of documents published per year. (b) Number of citations per year.

At present, domestic and foreign scholars have more and more research on portfolio optimization. It can be seen from the Citation Report retrieved from the relevant database that there is more and more literature research on portfolio optimization. The publication and citation of portfolio optimization literature are shown in Figure 1.

**Figure 2:**
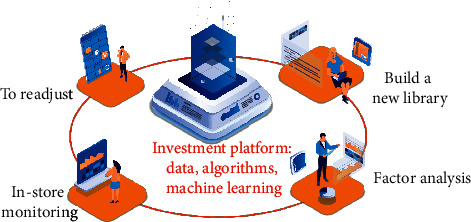
Financial investment of genetic algorithm.

After Kumar et al. put forward the portfolio theory, there have been many new portfolio developments: multiperiod portfolio, portfolio under different risk measures, portfolio based on transaction cost and liquidity, portfolio theory based on nonutility maximization, etc. [6, 7]. In the development history of portfolio theory, Ahn's work is the most prominent. By introducing a single factor model describing securities returns, it greatly simplifies the model of Markowitz theory and provides convenience for the successful application of Markowitz theory in the actual investment process. However, the theory is not perfect. Then Wang et al. proposed a multifactor model, which we call arbitrage pricing model [8]. Setiawan and Rosadi made an in-depth study on the securities price in the capital market by using statistical methods from both theoretical and empirical aspects in 1965 and put forward the efficient market theory [9]. The subsequent portfolio development is based on some of the above theories. In China, the research on portfolio can be traced back to the 1990s [10]. Of course, many valuable documents have emerged. Jiang et al. proposed a portfolio selection theory that strictly stated the operability under uncertain conditions: mean variance method, and conducted systematic, in-depth, and fruitful research. This theory led to the revolution of stock investment theory. Since Markowitz put forward the mean variance model, most portfolio models are based on the two parameters of mean and variance. With the development of portfolio theory, new risk measurement methods continue to emerge. Takahashi and Takahashi proposed the concept of semiabsolute deviation (Semia. D) and improved the mean variance model on this basis [11]. By analyzing the compound price of 50 representative stocks in Shanghai, it is concluded that the portfolio based on M-Semiad model is better than that in M-V model at each expected rate of return, and the effective portfolio satisfies the separation theorem of two funds. Finding the optimal solution of portfolio problem actually belongs to a kind of portfolio optimization problem, which usually comes down to quadratic programming model. The solving process is very cumbersome and requires high mathematical foundation, so many scholars use intelligent algorithms to solve it. Nahvi et al. applied binary coded genetic algorithm (GA) to solve this problem, which has the advantages of simplicity and intuition, but the efficiency is not high enough (Figure 2) [12]. Bakar and Rosbi applied integer coded adaptive genetic algorithm (AGA) to solve the problem, which improved the solution efficiency [13]. In 1964, Cheng and Zhong established the capital asset pricing model (CAPM) based on Markowitz. CAPM “accurately described the return and risk of assets” and studied the relationship between the expected return of assets and risky assets in the securities market and the formation of equilibrium price [14]. Pakistan proposed to solve the problem of portfolio optimization through symbiotic multiswarm PSO [15]. Mu and Xiong proposed portfolio analysis under background risk based on mean variance model [16]. Lin et al. put forward an adaptive portfolio model under new assets [17].

Based on the basic idea of ABC algorithm, this paper solves the cardinality constrained mean variance model (CCMV model) in portfolio problem. In the process of solving, a FABC algorithm which can always get the feasible solution is designed for CCMV model. Then the renewal equation of FABC is improved, and the IFABC algorithm with faster convergence speed is obtained. Finally, the IFABC algorithm is further improved based on quadratic programming, and a better QFABC algorithm is proposed.

## 3. Method

### 3.1. Quadratic Programming (QP)

Quadratic programming problem with inequality constraints, as shown in the following formulas:

(3) $$\begin{array}{c} \min\;q\left(x\right)=\frac{1}{2}x^{T}Hx+f^{T}x, \end{array}$$

(4) $$\begin{array}{c} \text{s.t.}\left(\begin{array}{c} Ax\leq b,\\ \text{Aeq}\cdot x=\text{beq},\\ lb\leq x\leq ub, \end{array}\right. \end{array}$$

*H*, *A*, and Aeq are matrices, *H* is a symmetric matrix of order *n*, and *f*, *b*, beq, *lb*, *ub*, and *x* are column vectors. *h* is a positive semidefinite matrix with a convex quadratic program or a nonconvex program. For convex quadratic programming, the objective function *q* (*x*) is a convex function. A quadratic programming strategy is the smallest solution in the world if it satisfies at least one *x* vector limit and has a lower limit in the potential range *q* (*x*). *H* is a positive definite matrix. If there is a globally optimal solution when there is a rigid convex quadratic program, it must be unique, as shown in the following equation:

(5) $$\begin{array}{c} \left[x,\text{fval,exitflag,output}\right]=\text{quadgrog}\left(H,f,A,b,\text{Aeq,beq},lb,ub,x_{0}\right). \end{array}$$

In general, this optimization strategy can be described as a convex quadratic programming strategy as shown in the following equation:

(6) $$\begin{array}{c} \underset{x\in R^{n}}{\min}\begin{array}{c} \sigma^{2}=\frac{1}{2}x^{T}Qx,\\ \mu^{T}x=\beta , \end{array}\\ \text{s.t.}\begin{array}{c} e^{T}x=\beta ,\\ x\geq 0. \end{array} \end{array}$$

This is a convex quadratic programming policy. Given the value of the parameter *β*, *β* is the expected return on investment with a uniquely optimal solution, as shown in the following equation:

(7) $$\begin{array}{c} x_{\mu }^{*}=\lambda Q^{-1}e+\gamma Q^{-1}\mu , \end{array}$$

where *λ*=*c* − *βb*/Δ, *γ*=*βa* − *b*/Δ, *a*=*e*^*T*^*Q*^−1^e, *c*=*μ*^*T*^*Q*^−1^*μ*,  and Δ=*ac* − *b*^2^.

The main problem of the above convex quadratic programming model is that the mean and covariance of assets are estimated from historical data and do not have sufficient accuracy. In fact, it is difficult to estimate the mean value of income, which is called mean ambiguity. In addition, the mean variance model is very sensitive to the distribution of input parameters, which will amplify the estimation error, lead to limit investment, and poor out of sample test results. Later, some improved Markowitz models were proposed to obtain stable investment. Later, in order to make the optimization process of investment more diversified, additional investment constraints were added to the model. For the investment allocation of minimizing risk, James Steiner estimation was proposed, and then robust estimation was proposed. In order to reduce transaction costs and the complexity of investment management, a class of important portfolio problems are proposed, which achieve this goal by limiting the amount of capital investment. Adding this constraint, the model is transformed into solving the sparse portfolio problem, which remedies the high instability of the traditional portfolio model. In particular, the Markowitz model is modified by adding constraints to control the number of assets [18]. This kind of problem is called the portfolio problem with cardinality constraints, as shown in the following equation:

(8) $$\begin{array}{c} \underset{x\in R^{n}}{\min}\begin{array}{c} \sigma^{2}=\frac{1}{2}x^{T}Qx,\\ \mu^{T}x=\beta , \end{array}\\ \text{s.t.}\begin{array}{c} {\left\|x\right\|}_{0}\leq K,\\ e^{T}x=1,\\ x\geq 0, \end{array} \end{array}$$

where ‖*x*‖_0_ is the zero norm of *x* and represents the number of nonzero elements in vector *x*; parameter *K* is the upper limit of the number of investment projects. In short, the modern portfolio problem mainly includes the following three objective functions:

1. Minimize investment risk *x*^*T*^*Qx*, min*x*^*T*^*Qx*
2. Maximize expected return on investment *μ*^*T*^*x*, max*μ*^*T*^*x*
3. Minimize the number of selected investment projects ‖x‖_0_, min‖*x*‖_0_

The restriction on the number of investment projects in the above cardinal constrained portfolio can be transformed into controlling the parameter *K* to a sufficiently small value; that is, the mathematical model of the financial portfolio problem mainly studied in this paper is obtained, as shown in the following equation:

(9) $$\begin{array}{c} \underset{x\in R^{n}}{\min}{\left\|x\right\|}_{0},\\ \mu^{T}x=\beta ,\\ s.t.x^{T}Qx\leq a,\\ e^{T}x=1,\\ x\geq 0, \end{array}$$

where *μ* is the rate of return of each asset, *Q* is the variance covariance matrix of each asset, *α* is the upper limit of acceptable risk of investors, and *β* is the expected rate of return of investors.

### 3.2. Genetic Algorithm

Genetic algorithm (GA) is a global optimization algorithm formed by simulating the evolution mechanism of natural selection and population “survival of the fittest” and “survival of the fittest.” Each possible problem solution is expressed as a “chromosome,” so as to obtain a “group” composed of chromosomes. This group is limited to the specific environment of the problem. Each individual is evaluated according to the predetermined objective function to obtain the individual fitness value. Individuals with higher adaptability to living environment often have higher survival probability. At the beginning, some individuals are always randomly generated, that is, candidate solutions. These individuals are cross combined by genetic algorithm according to the principle of “survival of the fittest” to produce offspring. The offspring inherit some excellent shapes of the parent generation, so they are obviously better than the previous generation. In this way, the population of “chromosome” will gradually evolve towards a better solution. Combined with genetic operations such as gene mutation in the process of species evolution, offspring more adapted to the environment may be produced [19].

Genetic algorithm is mainly composed of chromosome coding, initial population setting, fitness function setting, genetic operation (crossover, mutation) design, and so on. Coding refers to transforming the solution structure of practical problems into chromosome structure. Selection refers to selecting several individuals with higher fitness from the current population according to the selection probability and the fitness value of each chromosome. Fitness determines the individual's viability, usually roulette selection or tournament selection. Crossover refers to the mating recombination of some genes of two chromosomes according to the crossover probability and crossover strategy to produce new individuals. Crossover strategies generally include single point crossover and multipoint crossover. Mutation refers to the mutation of some genes in the chromosome according to the mutation probability and mutation strategy, which is another method for genetic algorithm to generate new individuals. For example, under the binary coding mode, the traditional mutation operation simply reverses the binary of the gene; that is, “0” becomes “1” and “1” becomes “0” [20].

The common parameters of genetic algorithm in China are shown in Table 1.

**Figure 3:**
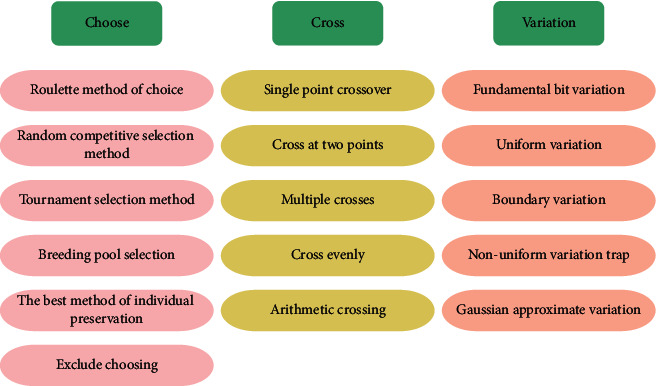
Operation method of genetic algorithm.

Genetic algorithms for solving optimization problems usually involve three basic operations: selection, crossover, and mutation. Selection is a key function of genetic algorithms. Different selection actions affect the speed of the genetic algorithm. Excessive selection pressure can improve the integration speed of the algorithm but can lead to early mergers. Therefore, it is necessary to choose the appropriate selection measure. Selection means selecting the most physically fit people and passing them on to the next generation with a higher probability and selecting the less physically fit people to pass on to the next generation. The selection process can bring the fitness value of individuals in the population closer to the optimal solution. Crossover operation is the process of replacing and reuniting parts of a parent's two personal structures to create a new individual. Crossover activity can create new people in next-generation populations and increase the efficiency of genetic algorithm search. Relying solely on the crossover operator is likely to bring you closer to local optimization. Mutation refers to the act of mutating an individual with a certain probability, and the value of the vector changes randomly among individuals with a low probability. Mutational activity creates a structure that is unprecedented in a population and increases the likelihood of approaching global optimization [21]. See Figure 3.

**Figure 4:**
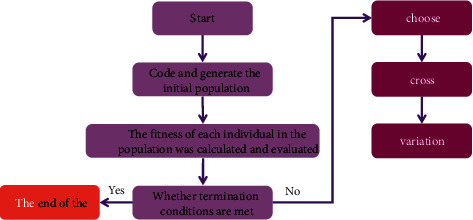
Flowchart of genetic algorithm.

The genetic algorithm that retains the optimal solution before or after selecting the operator can converge to the global optimal solution with probability 1. See Figure 4.

Execution steps of genetic algorithm:

1. Select an encoding strategy (binary encoding) and use an appropriate encoding strategy to represent each possible point in the problem search space, i.e., to create a chromosome.
2. Identify population size *n*, crossover and mutation methods, and genetic strategies such as genetic parameters such as selection probability PR, crossover probability PC, and mutation probability PM.
3. Set the number of iterations to *t* = 0, select a random chromosome to initiate the P (*o*) population, and determine the fitness function *f* (*F* > 0).
4. Calculate the fitness value for each chromosome.
5. The “best survival” process is implemented through a selection process to select better independent groups.
6. crossover operation on better chromosomes selected according to probability PC (single point crossover).
7. Participate in a mutation process with a PM probability for a gene on the chromosome.
8. The performance of the group determines whether it meets the predetermined termination criteria. Otherwise, return to 4.

### 3.3. Penalty Function Decomposition Method

In order to consider the financial portfolio problem, the general *l*_0_-minimization problem is accompanied by the fact that the *l*_0_-norm is part of the objective function or constraints.

Firstly, the first-order optimality condition of this kind of problem is given, and then the penalty function decomposition algorithm to solve this kind of problem is introduced; that is, the original problem is transformed into a series of penalty function subproblems, and the penalty function subproblems are solved by the block cooperative descent method, so as to obtain the solution of the original problem. It is proved theoretically that, under reasonable assumptions, the convergence point of the sequence obtained by the penalty function decomposition method satisfies the first-order optimality condition. In addition, *l*_0_ is the only nonconvex term in the original problem. It is proved that the convergence point is a local minimum point, and it can be proved that the convergence point of the sequence generated by the block cooperative descent method is the saddle point of the penalty function subproblem. Because *l*_0_ is the only nonconvex term, the convergence point is the local minimum of the penalty function subproblem.

Sparse problems are now widely used. For example, with a compressed sensor, it is possible to encode a large sparse signal with a relatively small number of linear dimensions, which converts the problem into a solution of a linear equation or a set of linear inequalities. Similar methods have been widely used in the field of linear regression. In recent years, the choice of sparse reverse covariance has become an important tool for finding conditional independent terms in image design. The current basic approach is to increase the log-probability function while finding a sparsely inverse covariance matrix. Similarly, the choice of characteristics for cluster problems suggests a promising approach to sparse logistic regression that seeks to reduce sparse logistics losses while seeking sparse solutions [22]. All the above applications can be expressed as the following *l*_0_ minimization problem, as shown in the following formulas:

(10) $$\begin{array}{c} \underset{x\in x}{\min}\left\{f\left(x\right):g\left(x\right)\leq 0,h\left(x\right)=0,{\left\|x_{J}\right\|}_{0}\leq r\right\}, \end{array}$$

(11) $$\begin{array}{c} \underset{x\in X}{\min}\left\{f\left(x\right)+\nu {\left\|x_{J}\right\|}_{0}:g\left(x\right)\leq 0,h\left(x\right)=0\right\}, \end{array}$$

where *r* > 0, *υ* > 0R > 0, *υ* > 0, and the sparsity of the problem is controlled by adjusting the size of *R* and *υ*. *X* is the closed convex set of *n*-dimensional Euclidean space *R*^*n*^. *g* : *R*^*n*^ ⟶ *R*, *g* : *R*^*n*^ ⟶ *R*^*m*^, *h* : *R*^*n*^ ⟶ *R*^*p*^ are continuously differentiable functions. ‖*x*_*J*_‖_0_ refers to the cardinality of the subvector marked by the index set *J* in *x*. Aiming at the special situation of this kind of problem, some algorithms are proposed. For example, iterative threshold method and matching pursuit method are developed to solve the *l*_0_-regularized least squares problem in compressed sensing, but they can not be used to deal with the general *l*_0_-minimization problem. A popular way to deal with the problem is to replace ‖•‖_0_ with *l*_1_ − norm‖•‖_1_ and then solve the relaxation problem. In applications such as compression sensing, problems can be solved under some reasonable assumptions. In recent years, some other relaxation methods have been proposed, that is, replacing *l*_0_ with *l*_*p*_. In general, the properties of the solutions of these methods are not very clear. The *l*_0_ regularization problem with upper and lower bound constraints is shown in the following equation:

(12) $$\begin{array}{c} \underset{x}{\min}\left\{f\left(x\right)+\lambda {\left\|x\right\|}_{0}:l\leq x\leq u\right\}. \end{array}$$

The hard threshold algorithm is proposed as follows, as shown in the following equation:

(13) $$\begin{array}{c} x^{k+1}\in \underset{x\in B}{\arg\min}\left\{f\left(x^{k}\right)+\nabla f{\left(x^{k}\right)}^{T}\left(x-x^{k}\right)+\frac{L}{2}{\left\|x-x^{k}\right\|}_{2}^{2}+\lambda {\left\|x\right\|}_{0}\right\}, \end{array}$$

where *λ* > 0, *L* > *L*_*f*_, *L*_*f*_ is the Lipschitz constant of ∇*f*(*x*), $B=\left\{x\in R^{n}:l\leq x\leq u\right\},l\in {\bar{R}}_{-}^{n},u\in \bar{R_{+}^{n}}$.

## 4. Experimental Results and Discussion

In this paper, we use a penalized decomposition method to solve this problem, i.e., a block cooperative method to solve the subproblem of the penalty function. According to some reasonable assumptions, the first point of the sequence formed by the penalized decomposition method provides a first-order optimization of the problem. Furthermore, when *h* is an affine function and *f* and *g* are convex functions, the join point is a local minimum of the problem. At the same time, the boundary points of the sequence formed by the joint collapse of the blocks are saddle points for the children of the penalty function. In addition, *h* is an affine function, *f* and *g* are convex functions, and the combination is a local minimum of the penalty function subproblem.

(14) $$\begin{array}{c} \underset{x\in X,y\in Y}{\min}\left\{f\left(x\right):g\left(x\right)\leq 0,h\left(x\right)=0,x_{J}-y=0\right\}, \end{array}$$

where *Y*={*y* ∈ *R*^*J*^ : ‖*y*‖_0_ ≤ *r*}.

The related quadratic penalty function is defined as follows, as shown in the following equation:

(15) $$\begin{array}{c} q_{p}\left(x,y\right)=f\left(x\right)+\frac{\rho }{2}\left({\left\|g{\left(x\right)}^{+}\right\|}^{2}+{\left\|h\left(x\right)\right\|}^{2}+{\left\|x_{J}-y\right\|}^{2}\right),\forall x\in X,y\in Y, \end{array}$$

where penalty function *ρ* > 0.

Now a penalty function decomposition method is proposed to solve the problem, which can be treated equivalently.

Let {*ε*_k_} be a decreasing sequence of positive terms. For the given *ρ*_0_ > 0, *σ* > 1, choose any *y*_0_^0^ ∈ *Y*, constant $\gamma \geq \max\left\{f\left(x^{\text{feas}}\right),\underset{x\in X}{\min}q_{po}\left(x,y_{0}^{0}\right)\right\},\text{make}k={}_{0^{{}^{\circ}}}$.

(1) Let *l*=0; apply the block cooperative descent method to solve the approximate solution of the penalty function subproblem through steps (a), (b), (c), and (d).
  (16) $$\begin{array}{c} \min\left\{q_{pk}\left(x,y\right):x\in X,y\in Y\right\}. \end{array}$$
  (a) Solve
    (17) $$\begin{array}{c} x_{l+1}^{k}\in \underset{x\in X}{\arg\min}q_{\rho k}\left(x,y_{l}^{k}\right). \end{array}$$
  (b) Solve
    (18) $$\begin{array}{c} y_{l+1}^{k}\in \underset{y\in Y}{\arg\min}q_{\rho k}\left(x_{l+1}^{k},y\right). \end{array}$$
  (c) Let (*x*^*k*^, *y*^*k*^)=(*x*_*l*+1_^*k*^, *y*_*l*+1_^*k*^), if (*x*^*k*^, *y*^*k*^) meets
    (19) $$\begin{array}{c} \left\|P_{\text{x}}\left(x^{k}-\nabla_{x}q_{\rho k}\left(x^{k},y^{k}\right)\right)-x^{k}\right\|\leq \varepsilon_{k}. \end{array}$$
  (d) Make*l* ← *l*+1, Turn to(a).
After analysis, the problem can be expressed equivalently, as shown in the following equation:
  (20) $$\begin{array}{c} \underset{x\in X,y\in R^{\left|J\right|}}{\min}\left\{f\left(x\right)+\nu {\left\|y\right\|}_{0}:g\left(x\right)\leq 0,h\left(x\right)=0,x_{J}-y=0\right\}. \end{array}$$
The related quadratic penalty function is defined as follows, as shown in the following equation:
  (21) $$\begin{array}{c} q_{\rho }\left(x,y\right)≔f\left(x\right)+\nu {\left\|y\right\|}_{0}+\frac{\rho }{2}\left({\left\|g{\left(x\right)}^{+}\right\|}^{2}+{\left\|h\left(x\right)\right\|}^{2}+{\left\|x_{J}-y\right\|}^{2}\right),\forall x\in X,y\in R^{\left|J\right|}. \end{array}$$
Included is penalty parameter *ρ* > 0.
Now a penalty function decomposition method is proposed to solve the problem, which can be treated equivalently. Let {*ε*_*k*_} be a decreasing sequence of positive terms for the given *ρ*_0_ > 0,*σ* > 1, *q*_0_. Choose any y_0_^0^ ∈ *Y*; constant *γ* satisfies $\gamma \geq \max\left\{f\left(x^{\text{feas}}\right)+\nu {\left\|x^{\text{feas}}\right\|}_{0},\underset{x\in X}{\min p_{\rho 0}\left(x,y_{0}^{0}\right)}\right\}$; let *k* = 0.
(2) Let *l*=0, the penalty function subproblem is solved by the block cooperative descent method described in steps (a)–(d), as shown in the following equation:
  (22) $$\begin{array}{c} \min\left\{p_{\rho k}\left(x,y\right):x\in X,y\in R^{\left|J\right|}\right\}. \end{array}$$
Approximate solution (*x*^*k*^, *y*^*k*^) ∈ *X* × *R*^|*J*|^.
  (a) Solve
    (23) $$\begin{array}{c} x_{l+1}^{k}\in \underset{x\in X}{\arg\min}q_{\rho k}\left(x,y_{1}^{k}\right). \end{array}$$
  (b) Solve
    (24) $$\begin{array}{c} y_{l+1}^{k}\in \underset{y\in R^{\left|J\right|}}{\arg\min}q_{\rho k}\left(x_{l+1}^{k},y\right). \end{array}$$
  (c) Let (*x*^*k*^, *y*^*k*^)=(*x*_*l*+1_^*k*^, *y*_*l*+1_^*k*^), if (*x*^*k*^, *y*^*k*^) meets
    (25) $$\begin{array}{c} \left\|P_{x}\left(x^{k}-\nabla_{x}q_{\rho k}\left(x^{k},y^{k}\right)\right)-x^{k}\right\|\leq \varepsilon_{k}. \end{array}$$
  Turn to (2).
  (d) Let *l* ← *l*+1; turn to (a);
  (1)  Let *ρ*_*k*+1_ : =*σρ*_*k*_;
  (2)  $\text{if}\underset{x\in X}{\min}p_{\rho k+1}\left(x,y^{k}\right)>\gamma ,\text{make}y_{0}^{k+1}:=x^{\text{feas}},\text{otherwise},\text{let}y_{0}^{k+1}=y^{k};$
  (3)  Let*k* ← *k*+1, turn to(1)_°_

**Figure 5:**
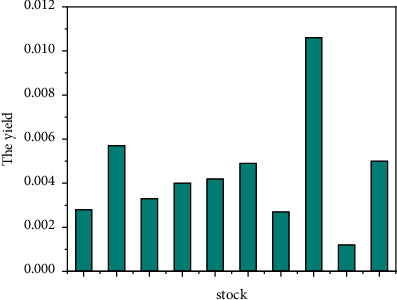
Average return of stocks.

**Figure 6:**
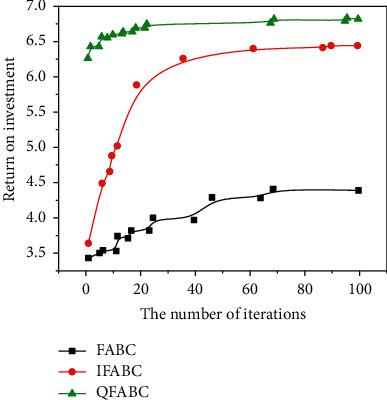
Comparison of three iterative algorithms.

This part mainly tests the penalty function decomposition method introduced earlier through numerical experiments, applies it to the portfolio model, and compares the results with the running results of genetic algorithm. Select the closing price (unit: yuan) of 10 constituent stocks in the 300 index for 100 trading days from January 5, 2019, to June 4, 2020. The data is from Sina Financial Data Center. The optimal asset allocation proportion of investors in these 10 constituent stocks is calculated by penalty function decomposition method and genetic algorithm [23]. See Figures 5 and 6.

**Figure 7:**
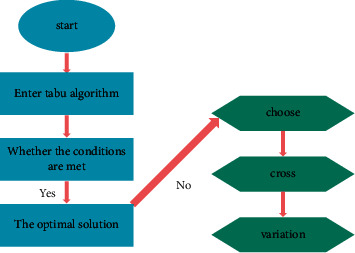
Analysis process of financial securities investment strategy.

The challenge of traditional analytical approaches to financial securities investment strategies needs to be addressed. During the analysis, it was found that the operational process of traditional strategic analysis methods is complex and the inability to solve the problem limits the scope of driving search [24]. Therefore, the advantage of the powerful local search capabilities of genetic algorithms is that they are used to search for parametric spaces. The process of analyzing the financial securities investment strategy based on a genetic algorithm is shown in Figure 7.

**Figure 8:**
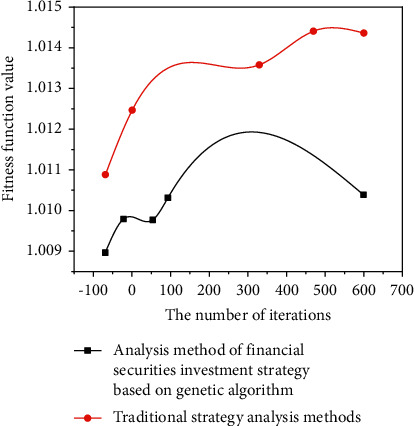
Experimental comparison results.

The results of the quadratic programming algorithm, the results of the Matlab programming solution of the genetic algorithm, the tools of the genetic algorithm, and the results of the solution are compared, focusing on the above Markovitz average variance model. See Figure 8.

In order to test the validity of the genetic algorithm method of financial portfolio analysis, a risk-free bank deposit in a particular city is selected and the real data is analyzed [25]. Select stocks in different sectors to invest in, taking into account diversification risks. There are four risky assets. To confirm the interpretation of the experiment, the traditional strategic analysis method is compared with the strategic analysis method of this model, and the final result is obtained by repeated experiments.

Finally, the penalty function decomposition method and the convergence solution of genetic algorithm are consistent. 67% of the total investment assets will buy a local automobile stock and 33% will buy a wine stock.

## 5. Conclusion

This paper proposes a genetic optimization algorithm to solve the financial portfolio problem. The quadratic programming algorithm (QP) and the penalty function decomposition algorithm are used to compare the three to solve the financial portfolio problem. Experimental results show that the quadratic programming algorithm (QP) has a faster convergence rate, but the investment distribution is not so good under the influence of the initial value selection. At the same time, the penalty function decomposition algorithm can be widely used in sparse financial portfolio problems, which ensures consistency with financial portfolio issues and provides effective value to the test results. Compared to the quadratic programming algorithm, the genetic algorithm is very good for global control, but not good enough for local search, so the late merger speed is reduced and decentralized investment is better than the quadratic programming algorithm. Thus, genetic algorithms are faster, more convenient, and more efficient in solving financial portfolio problems. However, because the data selected in the model is not as large as that in the actual financial portfolio problem, the complexity of the problem is greatly reduced. Therefore, in the future, quadratic programming algorithm and genetic algorithm can be applied to the financial portfolio of small-scale investment stocks.

## Figures and Tables

**Table 1:** Common parameters of genetic algorithm.

Control parameters	Meanings
String length (*L*)	The length of each code, including fixed length and variable length
Population capacity	Number of individuals per generation
Crossover rate	The probability of executing the crossover operator is recorded as PC
Variation rate	The probability of executing the mutation operator is recorded as PM

## Data Availability

The data used to support the findings of this study are available from the corresponding author upon request.
